# L166P mutant DJ-1 promotes cell death by dissociating Bax from mitochondrial Bcl-X_L_

**DOI:** 10.1186/1750-1326-7-40

**Published:** 2012-08-14

**Authors:** Haigang Ren, Kai Fu, Chenchen Mu, Xuechu Zhen, Guanghui Wang

**Affiliations:** 1 Department of Pharmacology, Laboratory of Molecular Neuropathology, Soochow University College of Pharmaceutical Sciences, Suzhou, Jiangsu, 215123, People’s Republic of China

**Keywords:** Parkinson’s disease, DJ-1, L166P, Mitochondria, Apoptosis, Bcl-X_L_, Bax, UVB

## Abstract

**Background:**

Mutations or deletions in DJ-1/PARK7 gene are causative for recessive forms of early onset Parkinson’s disease (PD). Wild-type DJ-1 has cytoprotective roles against cell death through multiple pathways. The most commonly studied mutant DJ-1(L166P) shifts its subcellular distribution to mitochondria and renders cells more susceptible to cell death under stress stimuli. We previously reported that wild-type DJ-1 binds to Bcl-X_L_ and stabilizes it against ultraviolet B (UVB) irradiation-induced rapid degradation. However, the mechanisms by which mitochondrial DJ-1(L166P) promotes cell death under death stimuli are largely unknown.

**Results:**

We show that DJ-1(L166P) is more prone to localize in mitochondria and it binds to Bcl-X_L_ more strongly than wild-type DJ-1. In addition, UVB irradiation significantly promotes DJ-1(L166P) translocation to mitochondria and binding to Bcl-X_L_. DJ-1(L166P) but not wild-type DJ-1 dissociates Bax from Bcl-X_L_, thereby leading to Bax enrichment at outer mitochondrial membrane and promoting mitochondrial apoptosis pathway in response to UVB irradiation.

**Conclusion:**

Our findings suggest that wild-type DJ-1 protects cells and DJ-1(L166P) impairs cells by differentially regulating mitochondrial Bax/Bcl-X_L_ functions.

## Background

Parkinson’s disease (PD), a common neurodegenerative movement disorder, is associated with progressive loss of dopaminergic neurons in the substantia nigra pars compacta (SNpc) [1]. DJ-1, a product of DJ-1/PARK7 gene, was originally identified as an oncogene protein that protects cells against stress through multiple pathways including gene transcription regulation, protein stabilization, signal transduction and reactive oxygen species (ROS) elimination [2,3]. Recently, DJ-1 has attracted more attention due to its involvement in familial early onset PD as its deletion mutants or point mutations including L166P, A104T, M26I, D149A, E64D and L10P cause PD [1,4,5]. DJ-1(L166P) was the most commonly studied and traditionally considered as “loss of function by degradation” because of its instability and misfolded structure compared with wild-type DJ-1 [6-8]. In addition, DJ-1(L166P) exists as a monomer, whereas wild-type DJ-1 exists as homodimers in cells [6,9]. However, many lines of recent evidence indicated that DJ-1(L166P) renders cells more susceptible to cell death under death stimuli [10-14].

Mitochondrial dysfunction is the key and common causative factor for pathogenesis of PD [1,15-19]. In PD patients and experimental PD models, dopaminergic neurodegeneration is caused at least partly by activation of mitochondria-dependent programmed cell death 2(PCD) pathways [20,21]. For instance, positive Bax, caspase-3, caspase-9 have been observed in SNpc dopaminergic neurons in PD models [22-24]. In addition, PD-associated proteins such as PINK1, parkin and DJ-1 directly affect mitochondrial functions [25-29]. DJ-1 deficiency leads to impairments of mitochondrial connectivity, fusion rates, membrane potential (ΔΨm), respiratory capacity and ROS scavenging [25,30-33]. Interestingly, wild-type DJ-1 partially localized in mitochondria and DJ-1 mutants including L166P are more prone to mitochondrial localization [5,8,12,34,35]. Moreover, both wild-type DJ-1 and DJ-1(L166P) are enriched in the mitochondrial fraction under death stimuli [34,36]. So, it is possible that DJ-1(L166P) impairs cells or neurons by “gain of function by translocation to mitochondria”. In addition, the physiological roles of their translocation to mitochondria under oxidative stress are still unclear because wild-type DJ-1 translocation to mitochondria under oxidative stress is required for its oxidation of Cys106 [34], but DJ-1(L166P) can not be oxidized [13], suggesting that these two proteins may differentially function in mitochondria.

Recently, we reported that wild-type DJ-1 translocates to mitochondria and binds to Bcl-X_L_ in response to UVB irradiation and inhibits Bcl-X_L_ rapid degradation and mitochondrial apoptosis pathway induced by UVB irradiation [37]. However, the roles of DJ-1(L166P) in mitochondria during oxidative stress are largely unknown. In this study, we further showed that DJ-1(L166P) binds more tightly to Bcl-X_L_ than wild-type DJ-1. Under UVB irradiation, DJ-1(L166P) translocates to mitochondria to dissociate Bax from Bcl-X_L_ by its interaction with Bcl-X_L_, resulting in an increased susceptibility of cells to UVB irradiation-induce cell death. Our results suggest that DJ-1 and DJ-1(L166P) differentially regulate Bcl-X_L_ functions in control of the mitochondrial apoptotic pathway.

## Results

### Subcellular distribution of wild-type DJ-1 and DJ-1(L166P)

**Figure 1:**
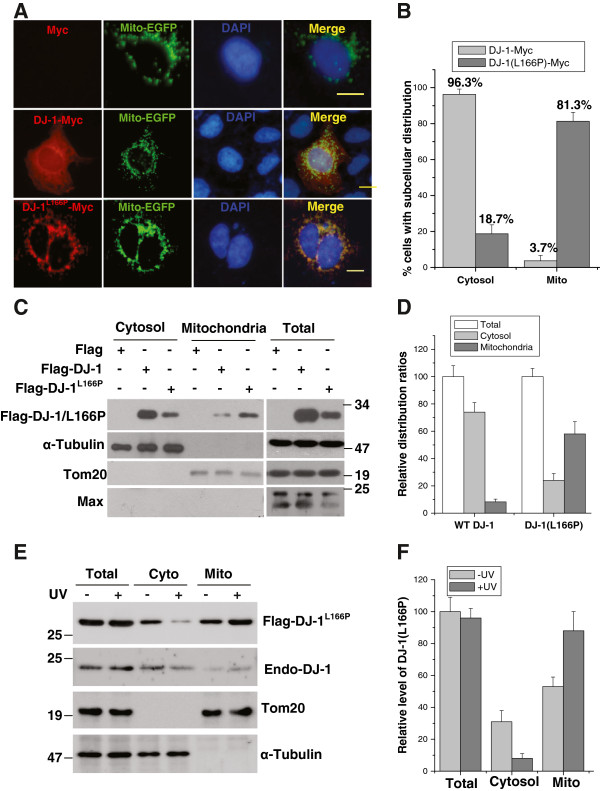
**Subcellular distribution of wild-type DJ-1 and DJ-1(L166P) in mitochondria.** (**A**) HEK293 cells transiently transfected with Myc (control), DJ-1-Myc or DJ-1(L166P)-Myc along with Mito-EGFP were subjected to immunocytochemical staining with anti-Myc antibodies (red) and then labeled with the nuclear marker DAPI (blue). Bar, 10 μm. (**B**) The percentage of cells transfected with DJ-1-Myc or DJ-1(L166P)-Myc presenting mitochondrial or cytosolic staining in (A) was quantified (mean ± S.E.M., n = 3; by one-way ANOVA). (**C**) H1299 cells transiently transfected with Flag, Flag-DJ-1 or Flag-DJ-1(L166P) were subjected to subcellular fractionation. The total cell lysate and the cytosolic and mitochondrial fractions were subjected to immunoblot analysis. (**D**) The distribution ratio of Flag-DJ-1 or Flag-DJ-1(L166P) in cytosolic or mitochondrial fractions to their total level in (C) were analyzed using densitometric analysis (mean ± S.E.M., n = 3; by one-way ANOVA). (**E**) H1299 cells transiently transfected with Flag-DJ-1(L166P) were treated with or without 80 mJ/cm^2^ UVB irradiation, and the cells were then subjected to the subcellular fractionation assay. The total cell lysate, cytosolic and mitochondrial fractions were subjected to immunoblot analysis. (**F**) The relative level of Flag-DJ-1(L166P) in (E) were analyzed using densitometric analysis (mean ± S.E.M., n = 3; by one-way ANOVA).

Considering that DJ-1 and its pathogenic mutant DJ-1(L166P) have potential functions in mitochondria, we first examined the subcellular localization of DJ-1 and DJ-1(L166P) in HEK293 cells. DJ-1-Myc was distributed diffusely in both the cytoplasm and nucleus, with a small portion co-localized with MitoTracker (green) (Figure 1A and 1B). However, DJ-1(L166P)-Myc was dominantly presented in the mitochondria with much less nuclear and cytosolic distribution (Figure 1A). Quantitative analysis showed that approximately 81.3% of cells transfected with DJ-1(L166P) displayed a mitochondrial localization, and approximately 18.7% of them displayed a cytosolic localization (Figure 1B). Consistent with the immunocytochemical results, subcellular fractionation assays also showed that both of distribution ratio and protein level of DJ-1(L166P) in the mitochondrial fraction were much higher than those of wild-type DJ-1, although the total protein level of DJ-1(L166P) was much less than that of wild-type DJ-1 (Figure 1C and 1D). The lower level of Flag-DJ-1(L166P) protein compared to Flag-DJ-1 should be caused by that L166P mutant is unstable and degraded rapidly through the ubiquitin proteasome system (UPS), when equal amounts of plasmids are used for transfection [6,7,38]. The mitochondrial localization of wild-type DJ-1 and its mutants increases under oxidative stresses such as paraquat treatment,H_2_O_2_ and UV irradiation [34,36]. Consistent with those findings, we observed that UVB irradiation increased the mitochondrial localization of both endogenous DJ-1 and Flag-DJ-1(L166P), but did not change total protein levels of them (Figure 1E and 1F). These results indicated that DJ-1(L166P) is prone to mitochondrial localization and the mitochondrial distribution of wild-type DJ-1 and DJ-1(L166P) are increased in response to UVB irradiation.

### Interactions between Bcl-X_L_ and DJ-1(L166P)

**Figure 2:**
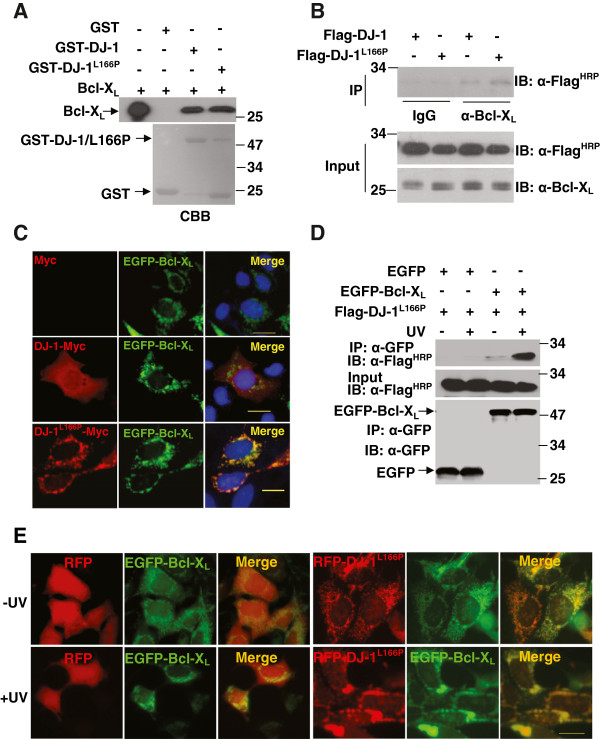
**Interactions between Bcl-X**_**L **_**and DJ-1(L166P).** (**A**) The supernatant of *E. coli* crude extract containing 50 μg of recombinant Bcl-X_L_ was incubated with 20 μg of GST, GST-DJ-1 or GST-DJ-1(L166P) coupled to glutathione-agarose beads. The bound proteins were detected by immunoblot analysis. (**B**) H1299 cells transiently transfected with Flag-DJ-1 or Flag-DJ-1(L166P) were subjected to immunoprecipitation with normal mouse serum or anti-Bcl-X_L_ antibody. (**C**) HEK293 cells transfected with Myc, DJ-1-Myc or DJ-1(L166P)-Myc along with EGFP-Bcl-X_L_ were subjected to immunocytochemical staining with anti-Myc antibodies (red) and then labeled with DAPI (blue). Bar, 10 μm. (**D**) H1299 cells transfected with Flag-DJ-1(L166P) along with EGFP or EGFP-Bcl-X_L_ were treated with or without 80 mJ/cm^2^ UVB irradiation. Supernatants of the cell lysates were then subjected to immunoprecipitation with anti-GFP antibodies. (**E**) HEK293 cells transiently transfected with RFP or DJ-1(L166P)-RFP along with EGFP-Bcl-X_L_ were treated with or without 80 mJ/cm^2^ UVB irradiation, and then the cells were observed with an inverted fluorescence microscope (Olympus, IX71). Bar, 10 μm.

In our previous study, we showed that wild-type DJ-1 translocates to mitochondria to bind to Bcl-X_L_ in response to UVB irradiation [37]. Considering that DJ-1(L166P) is mainly distributed in mitochondria, and translocates more to mitochondria under oxidative stress, we wonder whether DJ-1(L166P) binds to Bcl-X_L_. Although the interactions of wild-type DJ-1 and DJ-1(L166P) with Bcl-X_L_ were not significant different in GST pulldown assays *in vitro* (Figure 2A), more DJ-1(L166P) than wild-type DJ-1 bound to Bcl-X_L_ in cells (Figure 2B). Neither wild-type DJ-1 nor DJ-1(L166P) bound to Bcl_2_ and Bax, another two typical Bcl-2 family proteins (data not shown). These data suggested that wild-type DJ-1 and DJ-1(L166P) specifically bind to Bcl-X_L_. The monoclonal anti-Bcl-X_L_ antibody used in Figure 2B is suitable for immunoprecipitation assays as Flag-Bcl-X_L_ could be immunoprecipitated by anti-Bcl-X_L_ antibody but not by control mouse serum IgG ( Additional file 1: Figure S1). Consistent with data from immunoprecipitation analyses, immunocytochemical studies showed that DJ-1(L166P)-Myc, but not DJ-1-Myc, was well co-localized with EGFP-Bcl-X_L_ in HEK293 cells (Figure 2C). We also examined the interactions between Bcl-X_L_ and another pathogenic DJ-1 mutant, DJ-1(M26I). Similar to DJ-1(L166P), DJ-1(M26I) interacted with Bcl-X_L_ and co-localized with Bcl-X_L_ ( Additional file 1: Figure: S2). As DJ-1(L166P) increased in mitochondria under UVB irradiation (Figure 1E and 1F), we next performed immunoprecipitation assays to test if the interaction of Bcl-X_L_ with DJ-1(L166P) is affected by UVB irradiation. Interestingly, the binding affinity of Flag-DJ-1(L166P) for EGFP-Bcl-X_L_ significantly increased after UVB irradiation (Figure 2D). In addition, UVB irradiation led to larger punctate DJ-1(L166P)-RFP spots co-localizing with EGFP- Bcl-X_L_ (Figure 2E). Moreover, the mitochondria exhibited more severe abnormalities in cells harboring DJ-1(L166P) under UVB irradiation (Figure 2E).

### Requirement of the C-terminal of Bcl-X_L_ for DJ-1(L166P) binding

**Figure 3:**
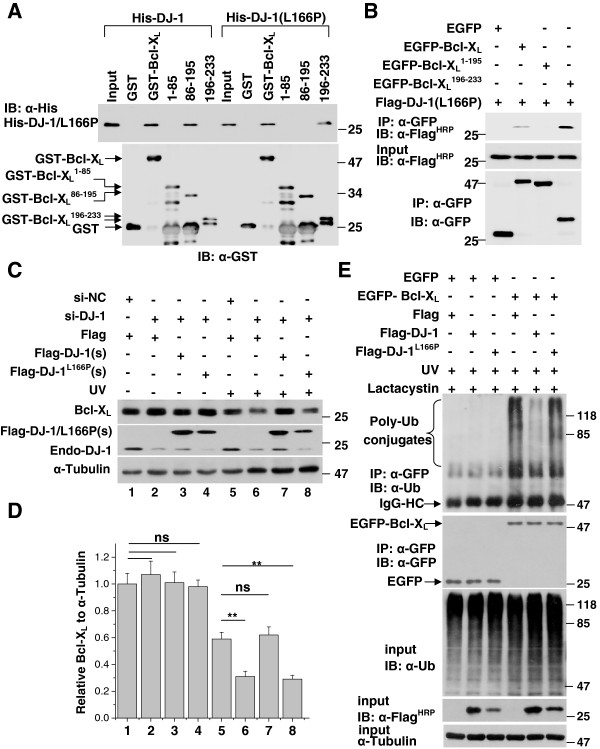
**Requirement of the C-terminus of Bcl-X**_**L **_**for DJ-1(L166P) binding.** (**A**) The supernatant of *E. coli* crude extract containing recombinant His-DJ-1 or His-DJ-1(L166P) was incubated with GST, GST-Bcl-X_L_, GST-Bcl-X_L_(1–85), GST-Bcl-X_L_(86–195) or GST-Bcl-X_L_(196–233) coupled to glutathione-agarose beads. The bound proteins were subjected to immunoblot analysis. (**B**) The supernatants of H1299 cells transfected with EGFP, EGFP-Bcl-X_L_, EGFP-Bcl-X_L_(1–195) or EGFP-Bcl-X_L_(196–233) along with Flag-DJ-1(L166P) were subjected to immunoprecipitation with anti-GFP antibodies. (**C**) H1299 cells transfected with si-NC or si-DJ-1 along with Flag, Flag-DJ-1(s) or Flag-DJ-1(L166P)(s) were treated with or without 80 mJ/cm^2^ UVB irradiation. Total cell lysates were subjected to immunoblot analysis. (**D**) The relative ratios of Bcl-X_L_ to α-Tubulin in (C) were analyzed using densitometry (mean ± S.E.M., n = 3; **, p <0.01; ns, no statistical significance by one-way ANOVA). (**E**) H1299 cells transfected with EGFP or EGFP-Bcl-X_L_ along with Flag, Flag-DJ-1 or Flag-DJ-1(L166P) were treated with 80 mJ/cm^2^ UVB and then with 5 μM Lactacystin for 16 hours. The supernatants of the cell lysates were subjected to immunoprecipitation with anti-GFP antibodies.

We previously found that wild-type DJ-1 mainly binds to amino acids 86–195 of Bcl-X_L_ which contain BH1, BH2 and BH3 domains [37]. We wonder whether DJ-1(L166P) binds to the same amino acids of Bcl-X_L_. Surprisingly, DJ-1(L166P) bound to the C-terminal fragment of Bcl-X_L_ at amino acids 196–233 (Figure 3A). We further examined the regions of Bcl-X_L_ binding to DJ-1(L166P) in H1299 cells. Similar to the results from GST pulldown assays, EGFP-Bcl-X_L_^196-233^ but not EGFP-Bcl-X_L_^1-195^ was found to interact with Flag-DJ-1(L166P) (Figure 3B). These results suggest that DJ-1(L166P) and wild-type DJ-1 bind to different domains of Bcl-X_L_, indicating that they may regulate Bcl-X_L_ functions differently. Under UVB irradiation, Bcl-X_L_ is degraded via the UPS [39,40]. In our previous study, we showed that wild-type DJ-1 stabilizes Bcl-X_L_ by its inhibiting Bcl-X_L_ under UVB irradiation. We therefore examined if DJ-1(L166P) also stabilize Bcl-X_L_. Under UVB irradiation, knockdown of DJ-1 decreased Bcl-X_L_ protein levels and re-overexpression of Flag-DJ-1(s), a synonymous mutant that is resistant to si-DJ-1, restored Bcl-X_L_ protein levels, however, Flag-DJ-1(L166P)(s) did not (Figure 3C and 3D). Meanwhile, the ubiquitination of Bcl-X_L_ was inhibited by DJ-1 but not DJ-1(L166P) (Figure 3E).

### Dissociation of Bax from Bcl-X_L_ by DJ-1(L166P)

**Figure 4:**
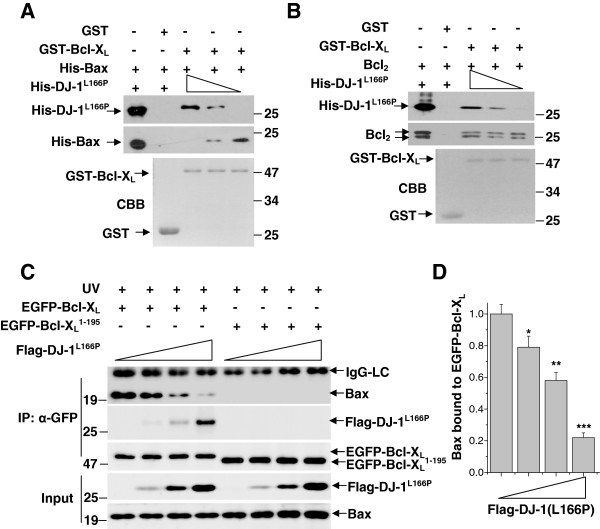
**Dissociation of Bax from Bcl-X**_**L **_**by DJ-1(L166P).** (**A**-**B**) Supernatant containing 50 μg of recombinant His-Bax (A) or Bcl_2_ (B) was incubated with 20 μg of GST or GST-Bcl-X_L_ coupled to glutathione-agarose beads. After being washed to remove unbound proteins, the glutathione-agarose beads were incubated with various amounts of His-DJ-1(L166P) supernatant. The bound proteins were subjected to immunoblot analysis. (**C**) H1299 cells stably expressing EGFP-Bcl-X_L_ or EGFP-Bcl-X_L_(1–195) were transiently transfected with various amounts of Flag-DJ-1(L166P) and treated with 80 mJ/cm^2^ UVB irradiation. The supernatants of cell lysates were subjected to immunoprecipitation with anti-GFP antibodies. (**D**) The relative levels of Bax bound to EGFP-Bcl-X_L_ in (D) were analyzed using densitometry (mean ± S.E.M., n = 3; *, p <0.05; **, p <0.01; ***, p <0.001; by one-way ANOVA).

Bcl-2 family proteins mediate apoptosis in a manner dependent on their homo- or hetero-dimerization [41]. Bcl-X_L_ interacts with Bax to block its oligomerization in the mitochondrial membrane, thereby protecting cells from Bax-induced mitochondrial membrane permeabilization [41-43]. It has been reported that the BH1-2 domains and the C-terminus of Bcl-X_L_ are required for Bcl-X_L_/Bax heterodimer formation [43,44]. To investigate if DJ-1(L166P) affects the interactions between Bcl-X_L_ and Bax or Bcl-2, we performed competitive binding assays. With less amount of His-DJ-1(L166P), more Bax bound to Bcl-X_L_ (Figure 4A). However, the binding ability of Bcl-2 to Bcl-X_L_ was not affected by His-DJ-1(L166P) (Figure 4B). To further identify if DJ-1(L166P) influences the interactions between Bcl-X_L_ and Bax in mammalian cells, we transfected various amounts of Flag-DJ-1(L166P) into H1299 cells stably expressing EGFP-Bcl-X_L_ or EGFP-Bcl-X_L_^1-195^ and performed immunoprecipitation assays. Under UVB irradiation, the amount of endogenous Bax that interacted with EGFP-Bcl-X_L_ was decreased when more Flag-DJ-1(L166P) was inputted (Figure 4C and 4D). However, EGFP-Bcl-X_L_^1-195^, which does not interact with Bax [44], was unable to interact with DJ-1(L166P) (Figure 4C).

### DJ-1(L166P) promotes cell death by interfering with Bcl-X_L_/Bax heterodimerization

The mitochondrial localization of Bax is important for its ability to induce cell death [41]. Because DJ-1 and DJ-1(L166P) re-distribute to mitochondria upon UVB irradiation but differentially influence Bcl-X_L_, we performed cytosolic and mitochondrial fractionation assays and MTT assays to examine the effects of DJ-1 and DJ-1(L166P) on mitochondrial Bax translocation and cell viability. We performed experiments in H1299 cells, a p53 null cell line to exclude the possibility that DJ-1 inhibits Bax transcription by binding to p53 [45-47]. Because endogenous DJ-1 expression is abundant [48], we constructed a H1299 cell line stably transfected with sh-DJ-1 to silence endogenous DJ-1 to examine the effects of exogenous wild-type DJ-1 and DJ-1(L166P).

**Figure 5:**
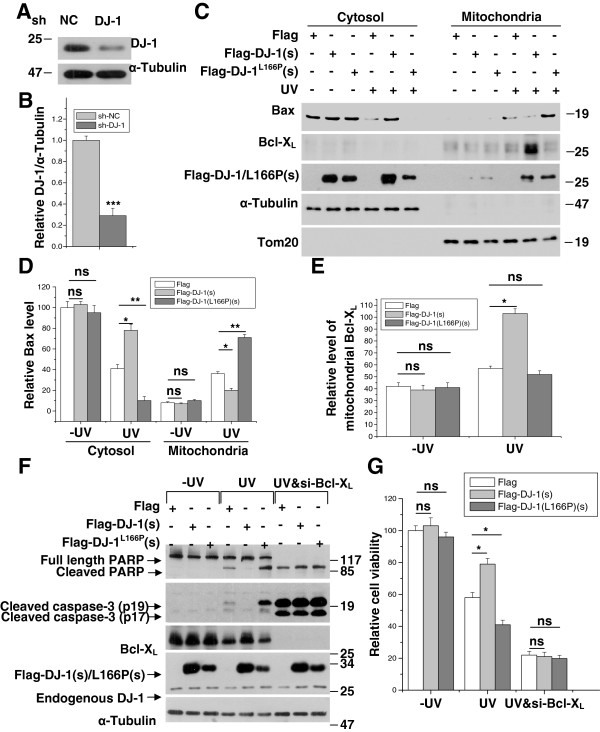
**Promotion of cell death by DJ-1(L166P)-influenced Bcl-X**_**L**_**/Bax heterodimerization.** (**A**) Cell lysates of H1299 cells stably expressing sh-NC or sh-DJ-1 were subjected to immunoblot analysis. (**B**) The relative ratios of DJ-1 to α-Tubulin in (A) were analyzed using densitometry (mean ± S.E.M., n = 3; ***, p <0.001; by one-way ANOVA). (**C**) H1299 cells stably expressing sh-DJ-1 were transfected with Flag, Flag-DJ-1(s) or Flag-DJ-1(L166P)(s) and treated with or without 80 mJ/cm^2^ UVB irradiation. The cells were then subjected to subcellular fractionation. (**D**) The relative level of Bax in (C) were analyzed using densitometry (mean ± S.E.M., n = 3; *, p <0.05; **, p <0.01; by one-way ANOVA). (**E**) The relative level of Bcl-X_L_ in mitochondria in (C) were analyzed using densitometry (mean ± S.E.M., n = 3; *, p <0.05; by one-way ANOVA). (**F**-**G**) H1299 cells stably expressing sh-DJ-1 transfected with si-NC or si-Bcl-X_L_ along with Flag, Flag-DJ-1(s) or Flag-DJ-1(L166P)(s) were treated with or without 80 mJ/cm^2^ UVB irradiation. Cell lysates were then subjected to immunoblot analysis (F) or the cells were analyzed with the MTT assay (G) (mean ± S.E.M., n = 3; *, p <0.05; ns, no statistical significance by one-way ANOVA).

The knockdown efficiency of sh-DJ-1 is shown in Figure 5A and 5B. In the absence of UVB irradiation, neither Flag-DJ-1(s) nor Flag-DJ-1(L166P)(s) had significant effects on the translocation of Bax from the cytosol to the mitochondria in sh-DJ-1 cells (Figure 5C and 5D). However, under UVB irradiation, less Bax was presented in the mitochondrial fraction in cells transfected with Flag-DJ-1(s), but more Bax was present in the mitochondrial fraction in cells transfected with Flag-DJ-1(L166P)(s) (Figure 5C and 5D). Overexpression of Flag-DJ-1(s) but not Flag-DJ-1(L166P)(s) significantly increased mitochondrial Bcl-X_L_ in response to UVB irradiation (Figure 5C and 5E). In addition, in the absence of death stimulus, overexpression of Flag-DJ-1(s) or Flag-DJ-1(L166P)(s) had no significant effects on Bcl-X_L_ levels, caspase-3 and PARP cleavage (Figure 5F) or cell viability (Figure 5G). However, with UVB irradiation, Flag-DJ-1(s) partially restored Bcl-X_L_ levels and accordingly inhibited the cleavage of caspase-3 and PARP (Figure 5F) and increased cell viability (Figure 5G). In contrast to Flag-DJ-1(s), Flag-DJ-1(L166P)(s) greatly increased the cleavage of both caspase-3 and PARP (Figure 5F) and decreased cell viability (Figure 5G). Moreover, the effects of DJ-1 and DJ-1(L166P) on cell death under UVB irradiation were abrogated by Bcl-X_L_ knockdown (Figure 5F and 5G). These results suggest that wild-type DJ-1 protects cells against UVB irradiation by inhibiting Bcl-X_L_ degradation, but DJ-1(L166P) promotes cell death by dissociating Bcl-X_L_/Bax heterodimerization.

## Discussion

Many studies have shown that wild-type DJ-1 and DJ-1(L166P) are partially localized in mitochondria [5,8,12,34,35], and that their mitochondrial distribution is enhanced under death stimuli [34,36]. Several lines of evidence indicate that wild-type DJ-1 exhibits its cytoprotective roles by maintaining mitochondrial integrity, fusion rates, membrane potential, respiratory capacity and ROS elimination [25,30-33].

In our previous study, we identified that wild-type DJ-1 is a novel partner of Bcl-X_L_ in mitochondria, to stabilize Bcl-X_L_[37]. Here, we found that DJ-1(L166P) binds to Bcl-X_L_ as well. However, DJ-1(L166P) does not stabilize Bcl-X_L_ but dissociates Bax from Bcl-X_L_. The binding ability of DJ-1(L166P) to Bcl-X_L_ is stronger than that of wild-type DJ-1 (Figure 2B). In contrast to DJ-1 that interacts with Bcl-X_L_ dependent on its oxidation [37], DJ-1(L166P) interacts with Bcl-X_L_ independent on its oxidation as DJ-1(L166P) is a loss of oxidized form [13]. In addition, DJ-1(L166P) binds to the C-terminus of Bcl-X_L_ (Figure 3A and 3B) which is required for Bcl-X_L_/Bax heterodimer formation [44], but wild-type DJ-1 mainly binds to middle regions (Figure 3A) containing BH1, BH2 and BH3 domains which are essential for Bcl-X_L_ stability [49,50]. Wild-type DJ-1 and DJ-1(L166P) that bind to different domains of Bcl-X_L_ may be due to the fact that L166P mutant interrupts the normal folding and exposes new domains or amino acid sites [38,51,52]. Taken together, our study suggests that the different roles of DJ-1 and DJ-1(L166P) in mitochondria may result from the different oxidative status of these two proteins and from their functioning differentially in mitochondria.

L166P mutant prevents normal folding of wild-type DJ-1 and itself is instable with a rapid degradation via UPS [6,7,51,52]. However, DJ-1(L166P) appears not only to be “loss of function” of wild-type DJ-1. It also forms larger complexes with other proteins but not wild-type DJ-1 [8]. Although DJ-1(L166P) loses the ability to bind to proteins that wild-type DJ-1 does, such as Daxx [53], DJ-1(L166P) existing as a monomer in cells may allow it to gain an ability to bind to proteins that wild-type DJ-1 does not. For instance, DJ-1(L166P) and DJ-1(M26I) bind more TTRAP than wild-type DJ-1 does, and they block the protective activity of TTRAP, leading to cell death [10]. Wild-type DJ-1 represses UV-induced JNK activation to protects cells, but DJ-1(L166P) significantly activates JNK pathway to promote cell death in response to UV irradiation [11]. As much more DJ-1(L166P) is translocated to mitochondria than wild-type DJ-1 under UVB stimulation, and DJ-1(L166P), but not wild-type DJ-1, dissociates Bax from mitochondrial Bcl-X_L_, it is therefore possible that DJ-1(L166P) may gain functions by translocation to mitochondria to affect mitochondrial pathway. We also found that another PD-associated mutant DJ-1(M26I) mainly distributes in mitochondria and binds to Bcl-X_L_, similar to DJ-1(166P) (Additional file 1: Figure S2). These results suggest that the mitochondrial Bcl-X_L_/Bax pathway influenced by mutant DJ-1 might be a common mechanism involved in mutant DJ-1-associated PD pathogenesis.

Mitochondrial dysfunction is a key feature involved in both sporadic and genetic forms of PD [1,15-19]. Although familial PD is rare, to understand the mechanisms and functions of familial PD-associated proteins in mitochondria may shed light on the pathogenesis of PD. Our findings suggest that wild-type DJ-1 and DJ-1(L166P) differentially mediate Bcl-X_L_ functions providing us to further understand the pathogenesis of PD.

## Conclusion

We found that a small portion of wild-type DJ-1 and most of DJ-1(L166P) is presented in mitochondria and wild-type DJ-1 and DJ-1(L166P) increased in mitochondria in response to UVB irradiation. DJ-1(L166P) binds to mitochondrial Bcl-X_L_ more tightly than wild-type DJ-1 and UVB irradiation further promotes their binding affinity. Unlike wild-type DJ-1, DJ-1(L166P) fails to stabilize Bcl-X_L_, but it dissociates Bax from Bcl-X_L_ that leading Bax enrichment in outer mitochondrial membrane and subsequently triggers cell death in response to UV irradiation. Our findings suggest that wild-type DJ-1 protects cells and DJ-1(L166P) impairs cells by differentially regulating Bcl-X_L_ functions. Our study provides a novel insight into the underlying mechanisms of PD pathogenesis.

## Materials and methods

### Cell culture and plasmid transfection

Human HEK293 cells, a human kidney cell line, and H1299 cells, a human lung cancer cell line, were maintained in DMEM (Dulbecco’s modified Eagle’s medium) supplemented with 10% fetal bovine serum (Hyclone, USA). Plasmid transfections were performed using Lipofectamine2000 reagent (Invitrogen, USA).

### UVB irradiation treatment

HEK293 or H1299 cells were irradiated with UVB using a UV crosslinker. Briefly, the cultured cells covered with a thin layer of phosphate buffer solution (PBS, pH 7.4), were exposed to UVB irradiation (312 nm) with 80 mJ/cm2 with a UV crosslinker (SCIENTZ03-II, Ningbo, China). After UVB irradiation, the cells were cultured for 16 hours and subsequently subjected to additional experiments.

### siRNA or shRNA knockdown

si-DJ-1^328^ was described previously [47]. siRNA against human Bcl-X_L_ mRNA was purchased from GenePharma (GenePharma, Shanghai, China) with the following sequences: sense: 5^′^-GAGAUGCAGGUAUUGGUGATT-3^′^, anti-sense: 5^′^-UCACCAAUACCUGCAUCUCTT-3^′^. The oligonucleotides were transfected with Oligofectamine reagent (Invitrogen, USA). Briefly, cultured cells were washed with Opti-MEM medium (Invitrogen) and then transfected with siRNA using Oligofectamine reagent in Opti-MEM medium without serum. Six hours after transfection, the culture medium was replaced with fresh complete medium. The cells were subjected to further experiments 72 hours after transfection. pGPU6/GFP/Neo-sh-DJ-1 encoding a short hairpin RNA (shRNA) against nucleotide 328 to 346 of human DJ-1 mRNA (sh-DJ-1) or a negative control short hairpin (sh-NC) was constructed by GenePharma (GenePharma, Shanghai, China). H1299 cells stably expressing sh-NC or sh-DJ-1 were obtained by selection with 200 μg/ml Geneticin (Invitrogen, USA) after transfection.

### Plasmid constructs

Full-length DJ-1 in p3 × Flag-myc-cmv-24, pET-15b, pDsRed-N1, pmyc-cmv-24 and pGEX-5x-1, and pET-21a-Bcl_2_, pET-21a-Bax, pET-21a-Bcl-X_L_, pEGFP-C2-Bcl-X_L_, p3 × Flag-myc-cmv-24-Bcl-X_L_, pGEX-5x-1-Bcl-X_L_(1–85), pGEX-5x-1-Bcl-X_L_(86–195) and pGEX-5x-1-Bcl-X_L_(196–233) were described previously [37]. pEGFP-C2-Bcl-X_L_(1–195) and pEGFP-C2-Bcl-X_L_(196–233) were created by subcloning PCR products into pEGFP-C2 at its *EcoR*I/*Sal*I sites. The PCR products were amplified with the following primers: 5^′^-CGGAATTCATGTCTCAGAGCAAC-3^′^ and 5^′^-GCGTCGACTCAATAGAGTTCCACAAA-3^′^ for 1-195aa; 5^′^-CGGAATTCGGGAACAATGCAGCA-3^′^ and 5^′^-GCGTCGACTCATTTCCGACTGAAG-3^′^ for 196-233aa. DJ-1(L166P) and DJ-1(M26I) mutants were obtained by site-directed mutagenesis using wild-type DJ-1 plasmids as template with the following primers: 5^′^-CCTGCAATTGTTGAAGCCCTGGAATG-3^′^ and 5^′^-CGCAAACTCGAAGCTGGTCCCAG-3^′^, for DJ-1(L166P); 5^′^-GTAGATGTCATTAGGCGA-3^′^ and 5^′^-GGTGACCTTAATCCCAGC-3^′^, for DJ-1(M26I); respectively. Two synonymous mutants, p3 × Flag-myc-cmv-24-DJ-1(s) and p3 × Flag-myc-cmv-24-DJ-1(L166P)(s), that are resistant to si-DJ-1^328^ and sh-DJ-1 were described previously [54].

### Immunocytochemistry

HEK293 cells were washed with PBS and fixed with 4% paraformaldehyde. After being blocked with 4% fetal bovine serum containing 0.25% Triton X-100 in PBS, the cells incubated with rabbit anti-myc polyclonal antibodies (Santa Cruz Biotechnology, USA) followed by an incubation with rhodamine-conjugated donkey anti-rabbit IgG (Santa Cruz biotech, Inc). After staining with DAPI (4^′^,6-diamidino-2-phenylindole), the labeled cells were observed using an inverted fluorescent microscope (Olympus, IX71).

### GST pulldown assay

Equal amounts of GST or GST-fused proteins (20 μg) expressed by Escherichia coli strain JM109 were incubated with 20 μl of glutathione agarose beads (Pharmacia, USA) for 30 min at 4°C. After washing three times with ice-cold PBS, the beads were incubated with 50 μg of His-fused protein expressed by Escherichia coli strain BL21 for 2 hours at 4°C. After incubation, the beads were washed five times with ice-cold HNTG buffer (20 mM Hepes-KOH, pH 7.5, 100 mM NaCl, 0.1% Triton X-100 and 10% glycerol). Bound proteins were eluted from the beads and subjected to immunoblot analysis with specific antibodies. The input represents 10% of the protein that was incubated with GST or a GST-fused protein. The inputs of purified GST and GST fusion proteins are stained with Coomassie Brilliant Blue (CBB) or anti-GST antibody.

### Immunoprecipitation assay

The cells were lysed in 1 ml cell lysis buffer (50 mM Tris–HCl pH 7.5 buffer containing 150 mM NaCl, 1% NP40 and 0.5% deoxycholate) supplemented with the protease inhibitor cocktail (Roche, USA) for 30 min at 4°C. After centrifugation at 12,000 g for 15 min at 4°C, the supernatants were incubated with appropriate antibodies coupled to protein G Sepharose (Roche, USA). The immunoprecipitants were then washed five times with cell lysis buffer. Bound proteins and cell lysates were subjected to immunoblot analysis. The input represents 10% of the supernatant used in the co-immunoprecipitation experiment.

### Immunoblot analysis and antibodies

Proteins were separated by 12% or 15% SDS-PAGE and subjected to immunoblot analysis with specific antibodies. The following primary antibodies were used: Monoclonal anti-Bcl2, anti-Bcl-XL, anti-GFP, anti-GST, anti-Tom20, anti-Ub and polyclonal anti-Myc, anti-Bax, anti-Max antibodies were purchased from Santa Cruz Biotechnology. Polyclonal anti-Bcl-XL, anti-cleaved caspase-3 and anti-PARP antibodies were from Cell Signaling. Polyclonal anti-DJ-1 antibodies were purchased from Chemicon. Monoclonal anti-Flag-HRP and anti-α-Tubulin antibodies were purchased from Sigma. Monoclonal anti-GAPDH antibody was from Millipore. The secondary antibodies, sheep anti-mouse IgG-HRP and anti-rabbit IgG-HRP were purchased from Amersham Pharmacia Biotech. The proteins were visualized using an ECL detection kit (Amersham Pharmacia Biotech). Immunoblot densitometric analysis of data from three independent experiments was performed using Photoshop 7.0 (Adobe, USA).

### Subcellular fractionation assay

The cytosolic and mitochondrial fractions were isolated using Mitochondria Isolation Kit for Cultured Cells (Beyotime, China). The total cell lysates and isolated fractions were subjected to immunoblot analysis with specific antibodies. Tom20, α-Tubulin and Max served as the mitochondrial, cytosolic and nuclear maker, respectively.

### Cell viability assay

The cell viability was measured by MTT (methylthiazoletetrazolium) assay. Briefly, the cells were washed with DMEM without phenol red (Gibco, USA) and incubated with 0.5 mg/ml MTT (Sigma) for three hours. The medium was removed and the formazan crystals were dissolved in DMSO (dimethyl sulfoxide). Cell viability was measured by spectrometry at OD570. The data were normalized to a control and the ratios are presented as means ± S.E.M from three independent experiments.

### Statistical analysis

The data were analyzed by one-way analysis of variance (ANOVA) using origin 6.0 software (Originlab, USA). Values are shown as mean ± S.E.M.

## Abbreviations

ANOVA: Analysis of variance; CBB: Coomassie brilliant blue; DAPI: 4^′^,6-diamidino-2-phenylindole; EGFP: Enhanced green fluorescent proteins; HRP: Horseradish peroxidase; PD: Parkinson’s disease; PBS: Phosphate buffer solution; ROS: Reactive oxygen species; SNpc: Substantia nigra pars compacta; UPS: Ubiquitin proteasome system; UVB: Ultraviolet B.

## Competing interests

The authors declare that they have no competing interests.

## Authors’ contributions

RHG designed and carried out all the experiments, and drafted the manuscript. FK and MCC participated in experiments and manuscript. ZXC edited the manuscript. WGH conceived and designed the study, and edited the manuscript. All authors read and approved the final manuscript.

## Supplementary Material

Additional file 1 **Figure S1. **Anti-Bcl-X_L_ antibody was suitable for immunoprecipitation. The supernatants of HEK293 cells that were transiently transfected with Flag-Bcl-X_L_ were subjected to immunoprecipitation analysis using normal mouse serum or anti-Bcl-X_L_ antibody. **Figure S2. **DJ-1(M26I) also interacted and co-localized with Bcl-X_L_ in cells. (A) HEK293 cells were co-transfected with EGFP or EGFP-Bcl-X_L_ along with Flag-DJ-1, Flag-DJ-1(L166P) or Flag-DJ-1(M26I) as indicated, the supernatants of cell lysates were subjected to immunoprecipitation analysis using anti-GFP antibodies. (B) HEK293 cells transiently transfected with DJ-1(M26I)-Myc with EGFP-Bcl-X_L_ were subjected to immunocytochemical staining with anti-Myc antibodies (red), Bar, 10 μm. Click here for file

## References

[B1] Moore DJ West AB Dawson VL Dawson TM Molecular pathophysiology of Parkinson’s disease Annu Rev Neurosci 2005 28 57 87 10.1146/annurev.neuro.28.061604.135718 16022590

[B2] Nagakubo D Taira T Kitaura H Ikeda M Tamai K Iguchi-Ariga SM Ariga H DJ-1, a novel oncogene which transforms mouse NIH3T3 cells in cooperation with ras Biochem Biophys Res Commun 1997 231 509 513 10.1006/bbrc.1997.6132 9070310

[B3] da Costa CA DJ-1: a newcomer in Parkinson’s disease pathology Curr Mol Med 2007 7 650 657 10.2174/156652407782564426 18045143

[B4] Guo JF Xiao B Liao B Zhang XW Nie LL Zhang YH Shen L Jiang H Xia K Pan Q Mutation analysis of Parkin, PINK1, DJ-1 and ATP13A2 genes in Chinese patients with autosomal recessive early-onset Parkinsonism Mov Disord 2008 23 2074 2079 10.1002/mds.22156 18785233

[B5] Bonifati V Rizzu P van Baren MJ Schaap O Breedveld GJ Krieger E Dekker MC Squitieri F Ibanez P Joosse M Mutations in the DJ-1 gene associated with autosomal recessive early-onset parkinsonism Science 2003 299 256 259 10.1126/science.1077209 12446870

[B6] Moore DJ Zhang L Dawson TM Dawson VL A missense mutation (L166P) in DJ-1, linked to familial Parkinson’s disease, confers reduced protein stability and impairs homo-oligomerization J Neurochem 2003 87 1558 1567 10.1111/j.1471-4159.2003.02265.x 14713311

[B7] Miller DW Ahmad R Hague S Baptista MJ Canet-Aviles R McLendon C Carter DM Zhu PP Stadler J Chandran J L166P mutant DJ-1, causative for recessive Parkinson’s disease, is degraded through the ubiquitin-proteasome system J Biol Chem 2003 278 36588 36595 10.1074/jbc.M304272200 12851414

[B8] Macedo MG Anar B Bronner IF Cannella M Squitieri F Bonifati V Hoogeveen A Heutink P Rizzu P The DJ-1L166P mutant protein associated with early onset Parkinson’s disease is unstable and forms higher-order protein complexes Hum Mol Genet 2003 12 2807 2816 10.1093/hmg/ddg304 12952867

[B9] Junn E Jang WH Zhao X Jeong BS Mouradian MM Mitochondrial localization of DJ-1 leads to enhanced neuroprotection J Neurosci Res 2009 87 123 129 10.1002/jnr.21831 18711745 PMC2752655

[B10] Zucchelli S Vilotti S Calligaris R Lavina ZS Biagioli M Foti R De Maso L Pinto M Gorza M Speretta E Aggresome-forming TTRAP mediates pro-apoptotic properties of Parkinson’s disease-associated DJ-1 missense mutations Cell Death Differ 2009 16 428 438 10.1038/cdd.2008.169 19023331

[B11] Mo JS Kim MY Ann EJ Hong JA Park HS DJ-1 modulates UV-induced oxidative stress signaling through the suppression of MEKK1 and cell death Cell Death Differ 2008 15 1030 1041 10.1038/cdd.2008.26 18309325

[B12] Shinbo Y Niki T Taira T Ooe H Takahashi-Niki K Maita C Seino C Iguchi-Ariga SM Ariga H Proper SUMO-1 conjugation is essential to DJ-1 to exert its full activities Cell Death Differ 2006 13 96 108 10.1038/sj.cdd.4401704 15976810

[B13] Taira T Saito Y Niki T Iguchi-Ariga SM Takahashi K Ariga H DJ-1 has a role in antioxidative stress to prevent cell death EMBO Rep 2004 5 213 218 10.1038/sj.embor.7400074 14749723 PMC1298985

[B14] Deeg S Gralle M Sroka K Bahr M Wouters FS Kermer P BAG1 restores formation of functional DJ-1 L166P dimers and DJ-1 chaperone activity J Cell Biol 2010 188 505 513 10.1083/jcb.200904103 20156966 PMC2828921

[B15] Winklhofer KF Haass C Mitochondrial dysfunction in Parkinson’s disease Biochim Biophys Acta 2010 1802 29 44 10.1016/j.bbadis.2009.08.013 19733240

[B16] Bueler H Impaired mitochondrial dynamics and function in the pathogenesis of Parkinson’s disease Exp Neurol 2009 218 235 246 10.1016/j.expneurol.2009.03.006 19303005

[B17] Martin I Dawson VL Dawson TM Recent advances in the genetics of Parkinson’s disease Annu Rev Genomics Hum Genet 2011 12 301 325 10.1146/annurev-genom-082410-101440 21639795 PMC4120236

[B18] de Moura MB dos Santos LS Van Houten B Mitochondrial dysfunction in neurodegenerative diseases and cancer Environ Mol Mutagen 2010 51 391 405 20544881 10.1002/em.20575

[B19] Henchcliffe C Beal MF Mitochondrial biology and oxidative stress in Parkinson disease pathogenesis Nat Clin Pract Neurol 2008 4 600 609 18978800 10.1038/ncpneuro0924

[B20] Perier C Bove J Vila M Mitochondria and programmed cell death in Parkinson’s disease: apoptosis and beyond Antioxid Redox Signal 2012 16 883 895 10.1089/ars.2011.4074 21619488

[B21] Vila M Przedborski S Targeting programmed cell death in neurodegenerative diseases Nat Rev Neurosci 2003 4 365 375 10.1038/nrn1100 12728264

[B22] Viswanath V Wu Y Boonplueang R Chen S Stevenson FF Yantiri F Yang L Beal MF Andersen JK Caspase-9 activation results in downstream caspase-8 activation and bid cleavage in 1-methyl-4-phenyl-1,2,3,6-tetrahydropyridine-induced Parkinson’s disease J Neurosci 2001 21 9519 9528 11739563 10.1523/JNEUROSCI.21-24-09519.2001 PMC6763046

[B23] Tatton NA Increased caspase 3 and Bax immunoreactivity accompany nuclear GAPDH translocation and neuronal apoptosis in Parkinson’s disease Exp Neurol 2000 166 29 43 10.1006/exnr.2000.7489 11031081

[B24] Hartmann A Michel PP Troadec JD Mouatt-Prigent A Faucheux BA Ruberg M Agid Y Hirsch EC Is Bax a mitochondrial mediator in apoptotic death of dopaminergic neurons in Parkinson’s disease? J Neurochem 2001 76 1785 1793 10.1046/j.1471-4159.2001.00160.x 11259496

[B25] Thomas KJ McCoy MK Blackinton J Beilina A van der Brug M Sandebring A Miller D Maric D Cedazo-Minguez A Cookson MR DJ-1 acts in parallel to the PINK1/parkin pathway to control mitochondrial function and autophagy Hum Mol Genet 2011 20 40 50 10.1093/hmg/ddq430 20940149 PMC3000675

[B26] McCoy MK Cookson MR DJ-1 regulation of mitochondrial function and autophagy through oxidative stress Autophagy 2011 7 531 532 10.4161/auto.7.5.14684 21317550 PMC3127213

[B27] Kamp F Exner N Lutz AK Wender N Hegermann J Brunner B Nuscher B Bartels T Giese A Beyer K Inhibition of mitochondrial fusion by alpha-synuclein is rescued by PINK1, Parkin and DJ-1 EMBO J 2010 29 3571 3589 10.1038/emboj.2010.223 20842103 PMC2964170

[B28] Cookson MR DJ-1, PINK1, and their effects on mitochondrial pathways Mov Disord 2010 25 Suppl 1 S44 S48 20187230 10.1002/mds.22713 PMC2840196

[B29] Van Laar VS Berman SB Mitochondrial dynamics in Parkinson’s disease Exp Neurol 2009 218 247 256 10.1016/j.expneurol.2009.03.019 19332061 PMC2752687

[B30] Irrcher I Aleyasin H Seifert EL Hewitt SJ Chhabra S Phillips M Lutz AK Rousseaux MW Bevilacqua L Jahani-Asl A Loss of the Parkinson’s disease-linked gene DJ-1 perturbs mitochondrial dynamics Hum Mol Genet 2010 19 3734 3746 10.1093/hmg/ddq288 20639397

[B31] Hao LY Giasson BI Bonini NM DJ-1 is critical for mitochondrial function and rescues PINK1 loss of function Proc Natl Acad Sci U S A 2010 107 9747 9752 10.1073/pnas.0911175107 20457924 PMC2906840

[B32] Blackinton J Lakshminarasimhan M Thomas KJ Ahmad R Greggio E Raza AS Cookson MR Wilson MA Formation of a stabilized cysteine sulfinic acid is critical for the mitochondrial function of the parkinsonism protein DJ-1 J Biol Chem 2009 284 6476 6485 19124468 10.1074/jbc.M806599200 PMC2649108

[B33] Ved R Saha S Westlund B Perier C Burnam L Sluder A Hoener M Rodrigues CM Alfonso A Steer C Similar patterns of mitochondrial vulnerability and rescue induced by genetic modification of alpha-synuclein, parkin, and DJ-1 in Caenorhabditis elegans J Biol Chem 2005 280 42655 42668 10.1074/jbc.M505910200 16239214 PMC3910375

[B34] Canet-Aviles RM Wilson MA Miller DW Ahmad R McLendon C Bandyopadhyay S Baptista MJ Ringe D Petsko GA Cookson MR The Parkinson’s disease protein DJ-1 is neuroprotective due to cysteine-sulfinic acid-driven mitochondrial localization Proc Natl Acad Sci U S A 2004 101 9103 9108 10.1073/pnas.0402959101 15181200 PMC428480

[B35] Zhang L Shimoji M Thomas B Moore DJ Yu SW Marupudi NI Torp R Torgner IA Ottersen OP Dawson TM Dawson VL Mitochondrial localization of the Parkinson’s disease related protein DJ-1: implications for pathogenesis Hum Mol Genet 2005 14 2063 2073 10.1093/hmg/ddi211 15944198

[B36] Blackinton J Ahmad R Miller DW van der Brug MP Canet-Aviles RM Hague SM Kaleem M Cookson MR Effects of DJ-1 mutations and polymorphisms on protein stability and subcellular localization Brain Res Mol Brain Res 2005 134 76 83 15790532 10.1016/j.molbrainres.2004.09.004

[B37] Ren H Fu K Wang D Mu C Wang G Oxidized DJ-1 Interacts with the Mitochondrial Protein BCL-XL J Biol Chem 2011 286 35308 35317 10.1074/jbc.M110.207134 21852238 PMC3186373

[B38] Olzmann JA Brown K Wilkinson KD Rees HD Huai Q Ke H Levey AI Li L Chin LS Familial Parkinson’s disease-associated L166P mutation disrupts DJ-1 protein folding and function J Biol Chem 2004 279 8506 8515 14665635 10.1074/jbc.M311017200

[B39] Zhang H Rosdahl I Bcl-xL and bcl-2 proteins in melanoma progression and UVB-induced apoptosis Int J Oncol 2006 28 661 666 16465371

[B40] Park K Lee JH Bcl-XL protein is markedly decreased in UVB-irradiated basal cell carcinoma cell lines through proteasome-mediated degradation Oncol Rep 2009 21 689 692 19212627

[B41] Youle RJ Strasser A The BCL-2 protein family: opposing activities that mediate cell death Nat Rev Mol Cell Biol 2008 9 47 59 10.1038/nrm2308 18097445

[B42] Yang E Zha J Jockel J Boise LH Thompson CB Korsmeyer SJ Bad, a heterodimeric partner for Bcl-XL and Bcl-2, displaces Bax and promotes cell death Cell 1995 80 285 291 10.1016/0092-8674(95)90411-5 7834748

[B43] Yin XM Oltvai ZN Korsmeyer SJ BH1 and BH2 domains of Bcl-2 are required for inhibition of apoptosis and heterodimerization with Bax Nature 1994 369 321 323 10.1038/369321a0 8183370

[B44] Jeong SY Gaume B Lee YJ Hsu YT Ryu SW Yoon SH Youle RJ Bcl-x(L) sequesters its C-terminal membrane anchor in soluble, cytosolic homodimers EMBO J 2004 23 2146 2155 10.1038/sj.emboj.7600225 15131699 PMC424420

[B45] Giaime E Sunyach C Druon C Scarzello S Robert G Grosso S Auberger P Goldberg MS Shen J Heutink P Loss of function of DJ-1 triggered by Parkinson’s disease-associated mutation is due to proteolytic resistance to caspase-6 Cell Death Differ 2010 17 158 169 10.1038/cdd.2009.116 19680261 PMC2796338

[B46] Bretaud S Allen C Ingham PW Bandmann O p53-dependent neuronal cell death in a DJ-1-deficient zebrafish model of Parkinson’s disease J Neurochem 2007 100 1626 1635 17166173 10.1111/j.1471-4159.2006.04291.x

[B47] Fan J Ren H Jia N Fei E Zhou T Jiang P Wu M Wang G DJ-1 decreases Bax expression through repressing p53 transcriptional activity J Biol Chem 2008 283 4022 4030 18042550 10.1074/jbc.M707176200

[B48] Bandopadhyay R Kingsbury AE Cookson MR Reid AR Evans IM Hope AD Pittman AM Lashley T Canet-Aviles R Miller DW The expression of DJ-1 (PARK7) in normal human CNS and idiopathic Parkinson’s disease Brain 2004 127 420 430 10.1093/brain/awh054 14662519

[B49] Feng Y Zhang L Hu T Shen X Ding J Chen K Jiang H Liu D A conserved hydrophobic core at Bcl-xL mediates its structural stability and binding affinity with BH3-domain peptide of pro-apoptotic protein Arch Biochem Biophys 2009 484 46 54 10.1016/j.abb.2009.01.003 19161970

[B50] Sattler M Liang H Nettesheim D Meadows RP Harlan JE Eberstadt M Yoon HS Shuker SB Chang BS Minn AJ Structure of Bcl-xL-Bak peptide complex: recognition between regulators of apoptosis Science 1997 275 983 986 10.1126/science.275.5302.983 9020082

[B51] Wilson MA Collins JL Hod Y Ringe D Petsko GA The 1.1-A resolution crystal structure of DJ-1, the protein mutated in autosomal recessive early onset Parkinson’s disease Proc Natl Acad Sci U S A 2003 100 9256 9261 10.1073/pnas.1133288100 12855764 PMC170905

[B52] Tao X Tong L Crystal structure of human DJ-1, a protein associated with early onset Parkinson’s disease J Biol Chem 2003 278 31372 31379 10.1074/jbc.M304221200 12761214

[B53] Junn E Taniguchi H Jeong BS Zhao X Ichijo H Mouradian MM Interaction of DJ-1 with Daxx inhibits apoptosis signal-regulating kinase 1 activity and cell death Proc Natl Acad Sci U S A 2005 102 9691 9696 10.1073/pnas.0409635102 15983381 PMC1172235

[B54] Ren H Fu K Mu C Li B Wang D Wang G DJ-1, a cancer and Parkinson’s disease associated protein, regulates autophagy through JNK pathway in cancer cells Cancer Lett 2010 297 101 108 10.1016/j.canlet.2010.05.001 20510502

