# Supplementary figures and images for: L166P mutant DJ-1 promotes cell death by dissociating Bax from mitochondrial Bcl-XL
Source: Mol Neurodegener. 2012 Aug 14;7:40. doi: 10.1186/1750-1326-7-40 (PMC3479024; doi:10.1186/1750-1326-7-40)

Additional File 1

Fig.S1

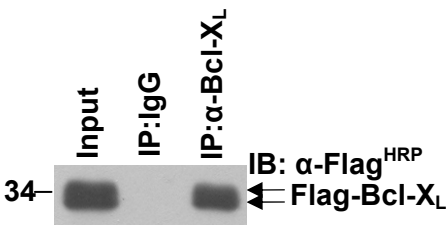

Fig.S2

A

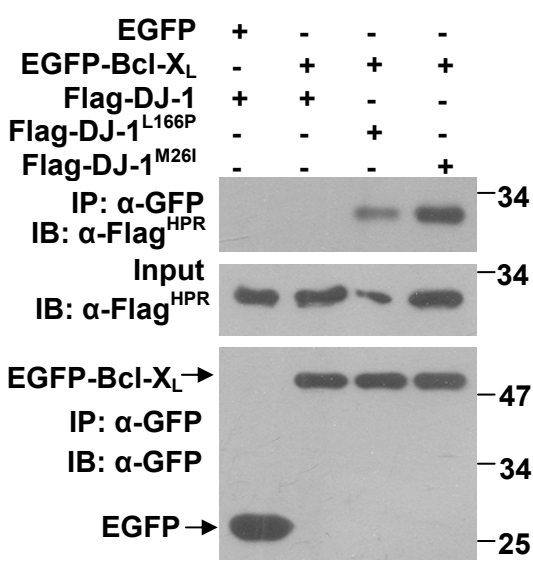

B

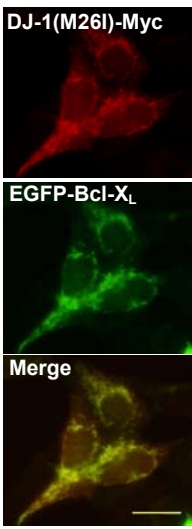

Supplement: Additional file 1 — Figure S1. Anti-Bcl-XL antibody was suitable for immunoprecipitation. The supernatants of HEK293 cells that were transiently transfected with Flag-Bcl-XL were subjected to immunoprecipitation analysis using normal mouse serum or anti-Bcl-XL antibody. Figure S2. DJ-1(M26I) also interacted and co-localized with Bcl-XL in cells. (A) HEK293 cells were co-transfected with EGFP or EGFP-Bcl-XL along with Flag-DJ-1, Flag-DJ-1(L166P) or Flag-DJ-1(M26I) as indicated, the supernatants of cell lysates were subjected to immunoprecipitation analysis using anti-GFP antibodies. (B) HEK293 cells transiently transfected with DJ-1(M26I)-Myc with EGFP-Bcl-XL were subjected to immunocytochemical staining with anti-Myc antibodies (red), Bar, 10 μm. [file 1750-1326-7-40-S1.pdf]
